# 대사증후군 환자의 관상동맥질환 관련 요인: 국민건강영양조사 자료 분석

**DOI:** 10.7586/jkbns.25.057

**Published:** 2025-11-24

**Authors:** Juhye Choi, Minjeong An

**Affiliations:** 1 Gradute school, College of Nursing, Chonnam National University, Gwangju, Korea; 1 전남대학교 간호대학 대학원; 2 College of Nursing, Chonnam National UniversityㆍChonnam Research Institute of Nursing Science, Gwangju, Korea; 2 전남대학교 간호학과

**Keywords:** Metabolic syndrome, Coronary artery disease, Risk factors, 대사증후군, 관상동맥질환, 관련요인

## Abstract

**Purpose:**

This study examined factors associated with coronary artery disease (CAD) among adults with metabolic syndrome using nationally representative survey data.

**Methods:**

A secondary analysis was conducted using data from the seventh (2016~2018) and eighth (2019~2021) Korea National Health and Nutrition Examination Surveys. Participants included 9,877 adults aged ≥ 19 years who met the National Cholesterol Education Program Adult Treatment Panel III criteria for metabolic syndrome. Complex sample analysis was applied, accounting for stratification, clustering, and weighting. Variables included sociodemographic, clinical, mental health, health behavior, and nutritional factors.

**Results:**

The prevalence of CAD among adults with metabolic syndrome was 5.6%. Older age, elevated systolic blood pressure, and higher total cholesterol were independently associated with CAD. Compared with adults aged 19~49 years, the adjusted odds ratios were 5.02 (95% confidence interval [CI] = 1.12~22.46) for those aged 50~59 years, 6.48 (95% CI = 1.16~36.04) for 60~69 years, and 7.77 (95% CI = 1.40~43.13) for ≥ 70 years. Participants with systolic blood pressure of 120~139 mmHg and ≥140 mmHg had 2.56 (95% CI = 1.10~5.95) and 3.69 (95% CI = 1.16~11.78) times higher odds, respectively. Total cholesterol levels of 200–239 mg/dL were associated with 3.65 times higher odds (95% CI = 1.13~11.78).

**Conclusion:**

Advanced age, elevated blood pressure, and borderline-to-high cholesterol were significantly associated with increased likelihood of CAD in adults with metabolic syndrome. These findings underscore the importance of early risk assessment and targeted preventive interventions for this high-risk population.

## 서론

### 1. 연구의 필요성

전 세계적으로 심혈관질환(cardiovascular disease)은 전체 사망의 약 3분의 1을 차지하는 대표적인 사망 원인으로[1], 우리나라에서도 암에 이어 두 번째로 높은 사망 원인으로 보고되고 있다[2]. 이 중 관상동맥질환(coronary artery disease, CAD)은 급성 심근경색과 협심증 등을 포함하는 주요 심혈관질환으로, 심장 근육에 혈액을 공급하는 관상동맥에 콜레스테롤 등의 지방 성분이 침착되어 혈관이 좁아지고, 혈류가 제한되면서 발생한다[3]. 관상동맥질환은 초기 증상이 뚜렷하지 않아 진단이 지연되며[4], 중증 상태에서 발견되는 경우가 많아 치명률이 높다[5]. 그러나 조기 발견 시 예후가 비교적 양호하며, 생활습관 관리를 통해 예방이 가능한 질환이므로, 고위험군의 조기 선별과 위험요인 관리는 중요한 공중보건 과제이다[6].

관상동맥질환의 고위험군 중 하나로는 대사증후군(metabolic syndrome)이 지목된다[7]. 대사증후군은 복부비만, 고혈압, 고혈당, 이상지질혈증 등 여러 대사 이상이 동시에 나타나는 상태로[8], 심혈관질환과 밀접히 연관되며 관상동맥질환의 주요 선행 질환으로 간주된다[9]. 우리나라 성인 중 약 21.3%가 대사증후군에 해당하며, 대사증후군의 구성요소 중 하나 이상 보유한 비율은 69.6%에 달한다[10]. 특히 남성, 60세 이상, 비활동적 생활습관을 가진 집단에서 뚜렷한 증가 추세를 보이고 있다[10]. 한편, 국외에서도 대사증후군은 전 세계 성인의 약 20%~25%가 해당되는 것으로 보고되며[11], 미국 국민건강영양조사(National Health and Nutrition Examination Survey)에서는 성인 대사증후군 유병률이 약 37%~42% 수준으로 나타났다[11,12]. 한국을 포함한 아시아 지역은 빠른 고령화, 식습관의 서구화, 비만 인구 증가, 신체활동 감소 등 생활양식 변화로 인해 향후 유병률이 더욱 증가할 것으로 전망된다[12]. 이러한 추세는 대사증후군이 특정 질환군을 넘어, 성인 인구 전반의 주요 만성질환으로 자리매김하고 있음을 시사한다. 더욱이, 대사증후군을 방치할 경우 전체 사망률은 1.35배, 심혈관질환 사망률은 1.74배, CAD 발생률은 1.52배로 증가한다[13]. 특히 대사 이상이 중첩될수록 관상동맥질환 위험이 기하급수적으로 증가하므로[14], 대사증후군은 예방을 위한 우선 개입 집단으로 고려해야 한다.

관상동맥질환의 주요 위험요인에는 고혈압, 고혈당, 이상지질혈증, 비만, 낮은 신체활동 수준, 흡연, 스트레스, 음주, 식습관 등이 있다[15]. 또한 연령, 성별, 교육 수준, 결혼 상태, 허리둘레, 과거 흡연력, 가족력 등 역시 독립적인 위험요인으로 제시되고 있다[16]. 이들 대부분은 대사증후군의 진단 기준과 일치하거나 유사하며, 복합적 요인이 존재할수록 발생 위험이 더 크다[14,17]. 즉, 대사증후군은 여러 위험요인을 동시에 지닌 ‘군집 위험 상태(clustering risk state)’로, 관상동맥질환의 고위험군에 해당한다[18]. 따라서 대사증후군 진단 항목 기반의 요인 분석은 임상 선별 전략과 공중보건 정책 수립의 근거가 될 수 있다.

기존 연구들은 대체로 대사증후군 또는 관상동맥질환 중 하나의 질환에 국한되거나, 특정 인구집단을 대상으로 한 단편적 연구가 많았다[7,19]. 또한 대부분 일부 건강행태나 생리적 지표에만 초점을 맞추어 다양한 위험요인의 상호작용을 종합적으로 분석하지 못한 한계가 있다[9,14]. 특히 인구사회학적 특성, 정신건강 상태, 식이섭취 등 다양한 요인을 통합적으로 반영한 관상동맥질환 이환 관련 분석은 매우 부족한 실정이다. 이에 대해 최근 종합 리뷰에서는 사회경제적 지위, 생활습관, 정신건강, 식이요소를 비롯한 다양한 사회적 결정요인과 생체지표를 결합한 분석이 거의 이루어지지 않았음을 지적하였다[20]. 이러한 배경에서, 전국 규모의 대표 표본조사인 국민건강영양조사(Korea National Health and Nutrition Examination Survey)는 인구사회학적 특성과 건강행태, 생리적 특성을 포함한 다변량 분석이 가능하다는 점에서, 대사증후군 내 관상동맥질환 이환 요인 규명에 유용하다.

본 연구는 국민건강영양조사 제7기(2016~2018)와 제8기(2019~2021) 자료를 활용하여, 성인 대사증후군 환자를 대상으로 관상동맥질환 이환에 영향을 미치는 요인을 포괄적으로 분석하고자 한다. 특히 인구사회학적 특성과 건강 관련 특성을 통합적으로 고려하여, 관상동맥질환 고위험군의 조기 선별에 필요한 근거를 제시하는 것이 목적이다. 이를 통해 대사증후군 환자의 건강위험 요인을 파악하고, 향후 예방 중심의 정책 수립을 위한 기초자료로 활용될 수 있을 것으로 기대된다.

### 2. 연구의 목적

1) 대사증후군 환자의 인구사회학적 특성, 건강관련특성, 관상동맥질환 이환 분포를 조사한다.

2) 대사증후군 환자의 인구사회학적 특성과 건강관련 특성에 따른 관상동맥질환 이환의 차이를 파악한다.

3) 대사증후군 환자의 관상동맥질환 이환에 영향을 미치는 관련 요인을 파악한다.

## 연구 방법

### 1. 연구 설계

본 연구는 대사증후군 환자의 관상동맥질환 관련 요인을 파악하기 위해 국민건강영양조사 제7기와 제8기 6차년도(2016~2021) 자료를 이차 분석한 서술적 조사 연구이다.

### 2. 연구 대상

본 연구는 국민건강영양조사 제7기(2016∼2018)와 제8기(2019∼2021)에 참여한 만 19세 이상 성인 29,230명 중, National Cholesterol Education Program Adult Treatment Panel III 기준(허리둘레는 대한비만학회 권고 적용)에 따라 대사증후군(5가지 구성요소 중 3가지 이상 해당)으로 진단된 9,877명을 대상으로 하였다[8,21]. 구성요소는 ① 수축기혈압(systolic blood pressure) ≥ 130 mmHg 또는 이완기혈압(diastolic blood pressure) ≥ 85 mmHg, 또는 고혈압 치료 중, ② 공복혈당(fasting blood sugar) ≥ 100 mg/dL 또는 혈당강하제 복용 중, ③ High-density lipoprotein (HDL) 콜레스테롤: 남성 < 40 mg/dL, 여성 < 50 mg/dL, 또는 이상지질혈증 치료 중, ④ 중성지방 ≥ 150 mg/dL 또는 이상지질혈증 치료 중, ⑤ 허리둘레: 남성 ≥ 90 cm, 여성 ≥ 85 cm였다.

임신·수유 중이거나 CAD 진단 여부 응답이 없는 경우는 제외하였다. 특히 임신·수유 중인 여성은 생리적 대사 변화와 복부 둘레 측정의 제한으로 인해 대사증후군 진단 기준 적용이 적절하지 않아 분석 대상에서 제외하였다[21,22].

### 3. 연구 도구

본 연구의 종속변수는 관상동맥 질환이며, 선행연구에서 관상동맥질환 관련 요인으로 나타난 변수 중 국민건강영양조사 자료에서 활용 가능한 변수를 활용하여 인구사회학적 특성, 건강관련 특성(질병관련요인, 정신건강요인, 건강행태 및 영양요인)으로 구분하여 사용하였다.

#### 1) 인구사회학적 특성

대상자의 인구 사회학적 특성으로 연령, 성별, 교육수준, 가구소득을 포함하였다. 연령은 19~49세, 50~59세, 60~69세, 70세 이상으로 구분하였고, 성별은 남과 여, 교육 수준은 초졸 이하, 중졸, 고졸, 대졸 이상으로 분류하였으며, 가구소득은 소득 4분위 기준(상, 중상, 중하, 하)을 상, 중, 하로 재분류하였다.

#### 2) 건강관련 특성

##### (1) 질병 관련 요인

질병 관련 요인은 수축기/이완기 혈압, 공복혈당, Hemoglobin A1c (HbA1c), 총콜레스테롤(total cholesterol), HDL-콜레스테롤, Low-density lipoprotein (LDL)-콜레스테롤, 중성지방, 체질량지수(Body mass index)를 포함하였다.

수축기 혈압은 정상(< 120 mmHg), 경계(120~139 mmHg, 고혈압 전단계), 위험(≥ 140 mmHg, 고혈압)으로, 이완기 혈압은 정상(< 80 mmHg), 경계(80~89 mmHg, 고혈압 전단계), 위험(≥ 90 mmHg, 고혈압)으로 분류하였다[23].

공복혈당은 정상(< 100 mg/dL), 경계(100~125 mg/dL, 공복혈당장애), 위험(≥ 126 mg/dL, 당뇨)으로, HbA1c는 정상(<5.7%), 경계(5.7~6.4%, 당뇨 전단계), 위험(≥ 6.5%, 당뇨)으로 분류하였다[8].

총콜레스테롤은 정상(< 200 mg/dL), 경계(200~239 mg/dL), 위험(≥ 240 mg/dL)으로 분류하였으며, LDL 콜레스테롤은 정상(≤ 100 mg/dL), 경계(100~129 mg/dL), 위험(≥ 130 mg/dL), 중성지방은 정상(< 150 mg/dL), 경계(150~500 mg/dL), 위험(≥ 500 mg/dL)으로 분류하였다[8].

HDL 콜레스테롤은 위험(< 40 mg/dL), 경계(41~59 mg/dL), 양호(≥ 60 mg/dL)로 분류하였다[8]. 체질량지수는 저체중(< 18.5 kg/m^2^), 정상(18.5~22.9 kg/m^2^), 과체중(23.0~24.9 kg/m^2^), 비만(≥ 25.0 kg/m^2^)으로 분류하였다[21].

##### (2) 정신건강 관련 요인

정신건강 관련 요인은 스트레스 인지율과 우울감 여부를 포함하였다. 스트레스 인지율은 ‘평소 느끼는 스트레스 정도’에 대한 응답을 ‘대단히 많이’ 또는 ‘많이 느끼는 편’은 많이 느낌, ‘조금 느낀다’ 또는 ‘거의 안 느낀다’는 적게 느낌으로 분류하였다. 우울감은 최근 1년 이내 2주 이상 지속된 슬픔 또는 절망감 경험 여부에 따라 예/아니오로 구분하였다.

##### (3) 건강행태 및 영양요인

건강행태 및 영양요인은 흡연, 고위험 음주, 신체활동량, 주중 하루 평균 좌식시간, 영양소 섭취량(총 에너지, 탄수화물, 단백질, 지방, 당, 나트륨)을 포함하였다.

흡연은 현재 흡연 여부, 고위험 음주는 주 2회 이상 음주하며 1회 남성 ≥ 7잔, 여성 ≥ 5잔인 경우로 정의하였다[22].

신체활동은 World Health Organization의 Global Physical Activity Questionnaire 지침에 따라 대사당량(Metabolic Equivalent of Task, MET)-min/week로 산정하였고, 고강도는 8.0 METs, 중강도 및 이동은 4.0 METs를 적용하여 낮음(< 600), 중등도(600–2,999), 높음(≥ 3,000)으로 분류하였다[24]. 좌식시간은 하루 평균 8시간 미만과 이상으로 구분하였다[25].

영양소 섭취량은 24시간 회상법 자료를 이용해 산출하였으며, 2020 한국인 영양소 섭취기준의 1일 에너지 기준 2,000 kcal와 나트륨의 만성질환 위험 감소 섭취량(2,300 mg)을 기준으로 평가하였다[26]. 하루 에너지 섭취량(< 2,000 kcal), 탄수화물(< 300 g), 단백질(< 54.6 g), 지방(< 67 g), 당(< 50 g), 나트륨(< 2,300 mg)은 적정, 그 이상은 과잉 섭취로 분류하였다.

#### 3) 관상동맥질환

관상동맥질환은 협심증 또는 심근경색증 중 하나 이상에 의사의 진단을 받은 경우 ‘있음’, 그렇지 않은 경우 ‘없음’으로 구분하였다.

### 4. 자료 수집

본 연구는 질병관리청에서 시행한 국민건강영양조사 원시자료를 이용하였다. 국민건강영양조사는 국민건강증진법 제16조에 근거한 전국 단위의 건강·영양조사로[22], 순환표본조사와 복합표본설계에 따라 전국 조사구와 가구를 2단계 층화집락표본추출 방법으로 선정하였다. 건강설문과 검진은 이동검진센터에서, 영양조사는 조사원이 가구를 직접 방문하여 시행하였다. 원시자료는 국민건강영양조사 홈페이지(https://knhanes.kdca.go.kr/knhanes)에서 승인절차를 거쳐 다운로드하였다.

### 5. 자료 분석

SPSS version 30.0 (IBM Corp., Armonk, NY, USA)을 사용하였으며, 통계적 유의수준은 *p* < .05로 설정하였다. 국민건강영양조사의 복합표본설계를 반영하기 위해 층화변수(kstrata), 집락변수(cluster), 가중치(weight)를 적용하여 복합표본분석을 시행하였고, 결측값은 유효한 값으로 처리하여 추정치의 표준오차가 과소 추정되지 않도록 하였다. 대상자의 인구사회학적 및 건강관련 특성, 관상동맥질환 이환 여부는 빈도와 가중 백분율로 제시하였다. 관상동맥질환 이환 차이는 Rao–Scott χ^2^검정을 사용하였으며, 유의한 변수는 복합표본 로지스틱 회귀분석에 포함하여 오즈비(odds ratio, OR)와 95% 신뢰구간(confidence interval, CI)으로 제시하였다.

### 6. 윤리적 고려

국민건강영양조사는 질병관리청 기관생명윤리위원회(IRB)의 승인을 받아 수행되었다(IRB No. 2018-01-03-5C-A, 2022-11-16-R-A). 본 연구는 전남대학교 생명윤리심의위원회에서 심의면제승인(IRB No. 1040198-241203-HR-199-01)을 받았다.

## 연구 결과

### 1. 대상자의 인구사회학적 특성에 따른 관상동맥질환 이환 차이

대상자의 인구사회학적 특성은 Table 1과 같다. 전체 대상자는 9,877명으로, 이 중 관상동맥질환 진단자는 551명(5.6%)이었다. 전체 대상자 9,877명 중 남성이 4,700명(56.8%), 여성이 5,177명(43.2%)이었으며, 관상동맥질환 진단군에서도 남성의 비율이 288명(58.3%)으로 더 높게 나타났으나 성별에 따른 유의한 차이는 없었다(χ^2^ = 0.42, *p* = .545).

연령은 19~49세가 2,222명(32.5%)으로 가장 많았고, 이어서 60~69세 2,672명(20.8%), 50~59세 1,921명(24.1%), 70세 이상 3,062명(22.6%) 순이었다. 반면 CAD 진단군에서는 70세 이상이 310명(51.8%)으로 가장 많았으며, 연령이 증가할수록 CAD 이환률이 유의하게 상승하였다 (χ^2^ = 324.50, *p* < .001).

교육 수준은 고등학교 졸업자가 2,815명(32.2%)으로 가장 많았고, 다음으로는 대학 이상 2,400명(31.4%), 초등학교 이하 3,294명(24.4%), 중학교 졸업 1,329명(12.0%) 순이었다. CAD 진단군에서는 초등학교 이하가 261명(42.9%)으로 가장 많아 교육 수준에 따른 유의한 차이를 보였다(χ^2^ = 109.81, *p* < .001).

소득 수준은 중간 소득군이 4,908명(51.1%)으로 가장 많았으며, 고소득군 2,166명(27.0%), 저소득군 2,780명(21.9%) 순이었다. CAD 진단군에서는 저소득군이 214명(33.5%)으로 가장 높게 나타났고, 소득 수준에 따른 CAD 진단 여부는 통계적으로 유의한 차이를 보였다(χ^2^ = 43.02, *p* < .001).

### 2. 대상자의 건강관련 특성에 따른 관상동맥질환 이환 차이

대상자의 건강 관련 특성은 Table 2과 같다. 수축기혈압 고위험군(≥ 140 mmHg)은 CAD 진단군에서 29.6%로 가장 높은 비율을 차지하였으며, 유의한 차이를 보였다(χ^2^ = 10.09, *p* = .010). 이완기혈압 정상군(< 80 mmHg)은 전체의 46.4%, CAD 진단군에서는 63.6%로, 통계적으로 유의한 차이를 보였다(χ^2^ = 63.04, *p* < .001).

공복혈당은 경계군(100~125 mg/dL)이 전체에서 54.2%로 가장 많았으며, CAD 진단군에서는 고위험군(≥ 126 mg/dL)이 28.4%로 유의하게 높았다(χ^2^ = 65.79, *p* < .001). HbA1c는 전체에서 경계군(5.7%~6.4%)이 가장 많았으나, CAD 진단군에서는 고위험군(≥ 6.5%)이 41.9%로 유의하게 높게 나타났다(χ^2^ = 24.34, *p* < .001).

총콜레스테롤은 정상군(< 200 mg/dL)이 CAD 진단군에서 88.6%로 가장 많았다(χ^2^ = 179.43, p < .001). 중성지방은 정상군(< 150 mg/dL)이 CAD 진단군에서 56.1%로 가장 많았다(χ^2^ = 52.68, *p* < .001). HDL은 집단 간 유의한 차이를 보이지 않았으며(χ^2^ = 0.55, *p* = .784), LDL은 정상군(< 130 mg/dL)이 CAD 진단군에서 74.0%로 높게 나타났다(χ^2^ = 62.41, *p* < .001).

체질량지수는 전체의 65.9%가 고위험군(≥ 25.0 kg/m^2^)에 속하였으나 CAD 진단군에서는 59.7%로 상대적으로 낮았다. 반면, 정상군(18.5~22.9 kg/m^2^)은 전체에서 13.4%였으나 CAD 진단군에서는 18.5%로 증가하여 통계적으로 유의한 차이를 보였다(χ^2^ = 14.73, *p* = .008).

정신건강 요인에서 스트레스 수준은 집단 간 유의한 차이를 보이지 않았다(χ^2^ = 1.44, *p* = .267). 그러나 우울감 경험은 CAD 진단군에서 16.3%로 높아 유의한 차이를 보였다(χ^2^ = 6.00, *p* = .028).

건강행태 및 영양 요인에서 현재 흡연율은 전체 22.3%였고, CAD 진단군에서는 17.2%로 낮았다(χ^2^ = 7.16, *p* = .013). 고위험 음주 비율은 CAD 진단군에서 17.1%로 낮았으나 통계적으로 유의하지 않았다(χ^2^ = 4.82, *p* = .063).

신체활동 수준은 낮은 활동군이 전체 64.2%, CAD 진단군에서 71.3%로 나타나 유의한 차이를 보였다(χ^2^ = 11.92, *p* = .004). 하루 평균 좌식 시간은 8시간 초과군이 전체 61.0%, CAD 진단군에서 66.1%로 높았다(χ^2^ = 5.13, *p* = .039).

하루 에너지 섭취량은 적정군(< 2,000 kcal)이 전체 61.4%, CAD 진단군에서 72.4%로 높게 나타났으며(χ^2^ = 25.68, *p* < .001), 단백질 섭취량은 적정군(< 54.6 g)이 CAD 진단군에서 50.3%로 유의하게 높았다(χ^2^ = 19.44, *p* < .001). 지방 섭취량은 적정군(< 67 g)이 CAD 진단군에서 90.5%로 가장 높았으며(χ^2^ = 17.97, *p* = .001), 나트륨 섭취는 과잉군(≥ 2,300 mg)이 전체 67.0%, CAD 진단군에서 59.5%로 유의하게 낮았다(χ^2^ = 12.39, *p* = .001).

### 3. 대상자의 관상동맥질환 이환 관련요인

대상자의 관상동맥질환 이환의 관련 요인은 Table 3과 같다. 단변량 분석에서 유의한 차이를 보인 변수들을 독립변수로 포함하여 복합표본 다중 로지스틱 회귀분석을 시행한 결과, 연령, 수축기 혈압, 총콜레스테롤이 유의한 관련 요인으로 확인되었다. 본 회귀모형의 설명력은 27.8%로 나타났으며(F = 25.18, *p* < .001, R^2^ = .28), 모형은 유의하게 적합하였다.

연령의 경우, 19~49세 대비 50~59세의 CAD 발생 가능성은 5.02배(OR = 5.02, 95% CI = 1.12, 22.46), 60~69세는 6.48배(OR = 6.48, 95% CI = 1.16, 36.04), 70세 이상은 7.77배(OR = 7.77, 95% CI = 1.40, 43.13) 높았다.

수축기 혈압은 정상군(< 120 mmHg)에 비해 고혈압 전단계군 (120~139 mmHg)에서 2.56배(OR = 2.56, 95% CI = 1.10, 5.95), 고혈압군(≥ 140 mmHg)에서 3.69배(OR = 3.69, 95% CI = 1.16, 11.78) CAD 발생 가능성이 높았다.

총콜레스테롤은 정상군(< 200 mg/dL) 대비 경계군(200~239 mg/dL)에서 3.65배(OR = 3.65, 95% CI = 1.13, 11.78) CAD 발생 가능성이 높았다.

## 논의

본 연구는 국민건강영양조사 자료를 활용하여 성인 대사증후군 환자의 관상동맥질환 이환 관련 요인을 분석한 결과, 주요 관련 요인은 연령, 수축기혈압, 총콜레스테롤로 확인되었다. 본 연구에서 대사증후군 환자의 CAD 이환율은 5.6%로 나타났으며, 이는 국내 한 선행연구에서 보고한 일반 성인의 CAD 유병률 3.16% 보다 약 1.8배 높은 수준이었다[27]. 이러한 결과는 대사증후군이 CAD 발생 위험을 약 2배 이상 증가시킨다고 보고한 선행연구 결과와 유사하며[7], 대사증후군 집단이 관상동맥질환의 고위험군임을 재확인하였다.

인구사회학적 요인에서는 연령, 교육수준, 소득수준에서 CAD 진단 여부에 따라 유의한 차이가 나타났으며, 성별에서는 차이가 없었다. 연령별 분포를 살펴보면, 70세 이상이 51.8%로 가장 높은 비율을 보였으며, 연령이 증가할수록 CAD 진단군의 분포가 뚜렷하게 높았다. 이러한 결과는 노화에 따른 혈관 내피세포 기능 저하, 누적된 죽상동맥경화, 동맥 경직 증가 등 복합적인 병태생리적 변화로 설명할 수 있으며[28,29], 선행연구에서도 연령 증가에 따라 심혈관질환 위험이 급격히 상승한다고 보고된 바 있다[15]. 교육수준과 소득수준이 낮을수록 CAD 분포율이 유의하게 높았는데, 이는 건강문해력 부족과 의료이용 접근성 저하로 인해 예방적 건강행동 실천이나 조기검진 참여가 제한될 가능성을 시사한다[14,20]. 이러한 결과는 사회경제적 요인이 CAD 발생에 영향을 미친다는 기존 연구결과와도 일치한다[20].

복합표본 로지스틱 회귀분석 결과, 연령, 수축기혈압, 총콜레스테롤이 CAD 이환과 유의한 관련성을 보였다. 연령은 CAD의 가장 강력한 예측 요인으로, 19~49세 대비 50~59세, 60~69세, 70세 이상에서 CAD 위험이 순차적으로 증가하였다. 선행연구에서도 심혈관질환 위험은 연령과 밀접하게 관련되어 10년마다 약 3배씩 증가하며[19], 특히 만 60세 이후에는 심혈관 사건 발생률이 급격히 상승하는 것으로 보고되었다[30].

수축기 혈압은 정상군(< 120 mmHg)에 비해 고혈압 전단계군(120~139 mmHg)부터 CAD 위험이 유의하게 증가하였다. 이는 혈압이 115/75 mmHg를 기준으로 20/10 mmHg 상승할 때마다 심혈관질환 위험이 두 배로 증가한다는 기존 연구를 뒷받침하며[31], 다기관 연구와 메타분석에서도 동일한 경향이 확인되었다[32,33]. 또한 수축기 혈압을 120 mmHg 이하로 집중 조절했을 때 표준 목표(140 mmHg 이하)보다 심혈관질환 발생 위험이 약 25% 감소했다는 임상시험 결과와도 맥락을 같이 한다[34]. 이러한 근거는 전고혈압 단계부터 적극적인 모니터링과 생활습관 교정이 필요함을 시사한다.

총콜레스테롤 또한 경계군(200~239 mg/dL)에서 CAD 위험이 정상군(< 200 mg/dL)보다 높았다. 이는 콜레스테롤 상승이 죽상동맥경화를 촉진하는 병태생리적 기전과 부합하며[35], 국내외 코호트 연구에서도 총콜레스테롤이 200 mg/dL 이상부터 심혈관질환 위험이 급격히 증가한다고 보고되었다[36]. 본 결과는 국내 이상지질혈증 관리 지침의 ‘총콜레스테롤 <200 mg/dL 유지 권고’와도 일관성을 보이며[37], 경계 수준에서도 정기적인 지질 모니터링이 필요함을 뒷받침한다.

본 연구는 기존 연구와 비교할 때 몇 가지 의의와 차별성을 갖는다. 복합표본 설계(가중치·층화·집락)를 반영하여 추정의 정확성과 대표성을 높였으며, 국민건강영양조사 2016~2021년의 최근 6개년 자료를 통합 자료를 활용함으로써 최신 경향을 반영하였다. 또한 기존 연구가 질병 진단 유무에 따라 집단을 단순 이분화한 것과 달리[27,38], 본 연구는 진단 전 단계를 별도로 구분하여 질병 단계별 위험 차이를 분석하였다. 그 결과, 경계 수준에서도 CAD 위험이 유의하게 증가함을 확인하여 조기 예방의 중요성을 실증적으로 제시하였다. 더불어 기존의 단일 요인 중심 연구와 달리[13,16], 인구사회학적 요인과 건강관련 요인을 통합적으로 고려하여 CAD 위험의 다차원적 구조를 규명하였고, 대사증후군이라는 특정 고위험군을 대상으로 질병 스펙트럼 전반을 포괄적으로 분석함으로써 조기 예방 중심의 전략 수립 근거를 마련하였다.

이러한 결과를 토대로 몇 가지 실무적·정책적 시사점을 제시할 수 있다. 첫째, 고령층에서는 만성질환 동반율이 높고 신체활동이 감소하여 CAD 위험이 가중되므로, 우리나라의 급속한 고령화 추세를 고려하여 연령 구조 변화에 대응한 조기 선별검사와 지속적 모니터링 체계를 구축해야 한다. 둘째, 경계 수준의 혈압(120~139 mmHg)과 총콜레스테롤(200~239 mg/dL)에 대해서도 조기 관리가 중요하다. 이 단계에서의 간호중재는 예방 효과가 크며, 간호사는 환자에게 위험성을 인식시키고 식이, 운동, 체중조절, 약물 순응 등 구체적인 자기관리 행동을 지도해야 한다. 간호사 주도의 맞춤형 교육은 대사증후군 고위험군의 생활습관 개선과 경계군의 혈압 및 지질 개선에 효과적인 것으로 보고되었다[39,40]. 또한 1차 의료기관과 보건소 간 연계관리체계를 구축하여 경계 수준의 혈압과 지질을 보이는 환자에게 즉시 생활습관 교육과 지속적 모니터링을 제공하는 것이 바람직하다. 이러한 통합관리 모델은 환자 추적관리의 효율성과 중재 지속성을 높이는 것으로 확인되었으며[41], 임상과 지역사회를 연계한 접근은 경계 단계에서의 위험인자 관리를 강화하고 CAD 고위험군의 조기 발견과 장기적 예방에 기여할 수 있다. 셋째, 정책적 차원에서 단계별 건강관리 서비스 연계 모델 구축이 필요하다. 현재 건강검진은 결과 통보에 그치는 경우가 많아, 경계군 대상자의 조기 개입이 제한적이다. 향후에는 검진 결과에 따라 상담 및 교육, 중재 서비스가 자동 연계되는 통합 관리 체계를 마련하고, 취약 지역을 중심으로 조기선별 및 등록관리 프로그램을 강화해야 한다. 이러한 통합적 접근은 대사증후군 환자의 경계 단계에서 위험 인식을 높이고 조기 중재를 강화하여 CAD 발생을 예방하며, 장기적으로 국민 건강 증진과 의료비 절감에 기여할 것이다.

본 연구는 전국 단위 자료를 활용하였다는 강점이 있으나, 횡단면 자료의 특성상 인과관계를 명확히 확정하기 어렵고, 자가보고형 변수에 따른 정보 편향의 가능성을 완전히 배제할 수 없다. 향후에는 장기 추적 코호트 연구를 통해 연령, 혈압, 지질 수준별 인과성을 검증하고, 맞춤형 중재 효과를 평가하는 연구가 필요하다. 이러한 후속 연구는 대사증후군 환자의 CAD 예방 전략을 보다 정밀하고 실효성 있게 설계하는 데 기여할 것이다.

## 결론

본 연구는 2016~2021년 국민건강영양조사 자료를 활용하여 성인 대사증후군 환자의 관상동맥질환 이환에 영향을 미치는 요인을 분석하였다. 그 결과, 연령, 수축기 혈압, 총콜레스테롤이 CAD와 유의하게 관련되었으며, 특히 고혈압 전단계(120~139 mmHg)와 총콜레스테롤 경계 수준(200~239 mg/dL)에서 CAD 위험이 증가하였다. 이러한 결과는 대사증후군 환자에서 연령 증가와 심혈관 위험인자 관리가 CAD 예방의 핵심임을 보여주며, 명확한 고위험군뿐만 아니라 경계 수준의 환자에서도 조기 개입의 필요성을 확인하였다.

이상의 연구결과를 바탕으로 다음과 같이 제언한다. 첫째, 대사증후군 환자의 연령 및 질병 경과에 따른 건강위험 요인의 변화 양상과 예측력을 확인할 수 있는 종단적 조사 연구를 제언하며, 둘째, 연령·혈압·지질 수준별로 위험도를 세분화하여 각 위험군에 최적화된 건강검진 전략과 생활습관 개선 프로그램을 개발하고, 그 효과를 검증하는 중재 연구를 제언한다. 셋째, 고혈압 전단계와 경계 수준의 콜레스테롤을 가진 대사증후군 환자를 대상으로 조기 개입 프로그램(식이·운동 중재, 약물치료 여부 검토 등)의 효과를 검증하는 임상시험 연구를 제언한다.

본 연구는 국가 단위 대표성을 갖춘 자료를 활용하여 대사증후군 환자의 CAD 위험요인을 규명하였다는 점에서 의의가 있으며, 향후 이러한 근거를 토대로 실질적인 1차 예방 전략과 맞춤형 건강관리 프로그램을 수립하는 데 기여할 것이다.

## Figures and Tables

**Table 1.** Coronary Artery Disease Distribution According to Sociodemographic Characteristics (N = 9,877, w^†^N = 12,247,157)

Variables	Categories	Total (n = 9,877)	Non-CAD (n = 9,326)	CAD (n = 551)	χ^2‡^	*p*
		n (w^†^%)	n (w^†^%)	n (w^†^%)		
Age (years)	19~49	2,222 (32.5)	2,210 (33.9)	12 (4.3)	324.50	< .001
	50~59	1,921 (24.1)	1,861 (24.5)	60 (16.0)		
	60~69	2,672 (20.8)	2,503 (20.4)	169 (27.9)		
	≥ 70	3,062 (22.6)	2,752 (21.2)	310 (51.8)		
Sex	Men	4,700 (56.8)	4,412 (56.8)	288 (58.3)	0.42	.545
	Women	5,177 (43.2)	4,914 (43.2)	263 (41.7)		
Educational status^§^	≤ Elementary school	3,294 (24.4)	3,033 (23.5)	261 (42.9)	109.81	< .001
	Middle school	1,329 (12.0)	1,259 (11.8)	90 (15.1)		
	High school	2,815 (32.2)	2,693 (32.6)	122 (23.9)		
	≥ College	2,400 (31.4)	2,323 (32.1)	77 (18.1)		
Income level^§^	Low	2,780 (21.9)	2,566 (21.3)	214 (33.5)	43.02	< .001
	Middle	4,908 (51.1)	4,656 (51.3)	252 (47.6)		
	High	2,166 (27.0)	2,081 (27.4)	85 (18.9)		

All complex sample analyses were performed including missing values treated as valid cases. CAD = Coronary artery disease; Non-CAD = No coronary artery disease. ^†^w= Weighted sample; ^‡^Rao–Scott χ^2^ test for complex sample design; ^§^Variables include missing data in the analysis.

**Table 2.** Coronary Artery Disease Distribution According to Health-related Characteristics (N = 9,877, w^†^N = 12,247,157)

Variables	Categories	Total (n = 9,877)	Non-CAD (n = 9,326)	CAD (n = 551)	χ^2‡^	*p*
		n(w^†^%)	n (w^†^%)	n (w^†^%)		
Disease-related factors						
SBP (mmHg)^§^	Normal (< 120)	2,534 (27.4)	2,405 (27.6)	129 (23.4)	10.09	.010
	Borderline (120~139)	4,732 (48.8)	4,484 (48.9)	248 (47.0)		
	High-risk (≥ 140)	2,602 (23.8)	2,428 (23.5)	174 (29.6)		
DBP(mmHg)^§^	Normal (< 80)	5,147 (46.4)	4,780 (45.5)	367 (63.6)	63.04	< .001
	Borderline (80~89)	3,057 (33.0)	2,925 (33.4)	132 (26.0)		
	High-risk (≥ 90)	1,664 (20.6)	1,612 (21.1)	52 (10.4)		
FBS (mg/dL)	Normal (< 100)	2,642 (26.6)	2,506 (26.8)	136 (23.5)	65.79	< .001
	Borderline (100~125)	5,211 (54.2)	4,943 (54.5)	268 (48.1)		
	High-risk (≥ 126)	2,024 (19.2)	1,877 (18.7)	147 (28.4)		
HbA1c (%)^§^	Normal (< 5.7)	2,750 (31.5)	2,664 (32.3)	86 (15.0)	24.34	< .001
	Borderline (5.7~6.4)	4,591 (45.3)	4,348 (45.5)	243 (43.1)		
	High-risk (≥ 6.5)	2,515 (23.2)	2,294 (22.2)	221 (41.9)		
TC (mg/dL)	Normal (< 200)	6,119 (58.7)	5,638 (57.2)	481 (88.6)	179.43	< .001
	Borderline (200~239)	2,630 (28.4)	2,580 (29.4)	50 (8.2)		
	High-risk (≥ 240)	1,128 (12.9)	1,064 (13.4)	64 (3.2)		
TG (mg/dL)	Normal (< 150)	4,532 (40.9)	4,211 (40.2)	321 (56.1)	52.68	< .001
	Borderline (150~499)	2,479 (25.5)	2,348 (25.6)	131 (23.4)		
	High-risk (≥ 500)	2,866 (33.6)	2,767 (34.2)	99 (20.5)		
HDL (mg/dL)^§^	Normal (≥ 60)	3,695 (39.9)	3489 (39.9)	206 (40.4)	0.55	0.784
	Borderline (41~59)	5,244 (51.9)	4950 (51.9)	294 (52.4)		
	High-risk (< 40)	909 (8.2)	862 (8.2)	97 (7.2)		
LDL (mg/dL)^§^	Normal (< 130)	1,047 (33.9)	978 (32.7)	69 (74.0)	62.41	< .001
	Borderline (130~159)	901 (32.5)	881 (33.0)	20 (18.8)		
	High-risk (≥ 160)	919 (33.6)	909 (34.3)	10 (7.2)		
BMI (kg/m^2^)^§^	Underweight (< 18.5)	52 (0.4)	52 (0.4)	0 (0.0)	14.73	.008
	Normal (18.5~22.9)	1,489 (13.4)	1,389 (13.2)	100 (18.5)		
	Overweight (23.0~24.9)	2,139 (20.3)	2,016 (20.2)	123 (21.8)		
	Obese (≥ 25.0)	6,114 (65.9)	5,793 (66.2)	321 (59.7)		
Mental health factors						
Stress^§^	Low	7,451 (73.6)	7,033 (73.5)	418 (76.0)	1.44	.267
	High	2,402 (26.4)	2,270 (26.5)	132 (24.0)		
Depressive symptom^§^, Yes		630 (11.5)	582 (11.2)	48 (16.3)	6.00	.028
Lifestyle behaviors and nutritional intake						
Current smoker^§^, Yes		1,770 (22.3)	1,683 (22.5)	87 (17.2)	7.16	.013
High-risk drinking^§^, Yes		1,248 (22.8)	1,200 (23.0)	48 (17.1)	4.82	.063
Physical activity level	Low	6,594 (64.2)	6,197 (63.8)	397 (71.3)	11.92	.004
	Moderate	2,992 (32.4)	2,850 (32.7)	142 (26.9)		
	High	291 (3.4)	279 (3.5)	12 (1.8)		
Sitting time (hours)^§^	Low (< 8)	3,935 (39.0)	3,745 (39.2)	190 (33.9)	5.13	.039
	High (≥ 8)	5,720 (61.0)	5,367 (60.8)	353 (66.1)		
Energy intake^§^ (kcal)	Adequate (< 2,000)	6,564 (61.4)	6,158 (60.9)	406 (72.4)	25.68	< .001
	Excessive (≥ 2,000)	3,300 (38.6)	3,155 (39.1)	145 (27.6)		
Carbohydrate intake (g)^§^	Adequate (< 300)	6,165 (60.9)	5,811 (60.7)	354 (64.2)	2.25	.168
	Excessive (≥ 300)	3,699 (39.1)	3,502 (39.3)	197 (35.8)		
Protein intake (g)^§^	Adequate (< 54.6)	4,526 (40.5)	4,229 (40.0)	297 (50.3)	19.44	< .001
	Excessive (≥ 54.6)	5,338 (59.5)	5,084 (60.0)	254 (49.7)		
Fat intake (g)^§^	Adequate (< 67)	8,605 (83.4)	8,097 (83.0)	508 (90.5)	17.97	.001
	Excessive (≥ 67)	1,259 (16.6)	1,216 (17.0)	43 (9.5)		
Sugar intake (g)^§^	Adequate (< 50)	5,483 (53.4)	5,165 (53.2)	318 (56.9)	2.38	.165
	Excessive (≥ 50)	4,381 (46.6)	4,148 (46.8)	233 (43.1)		
Sodium intake (mg)^§^	Adequate (< 2,300)	3,648 (33.0)	3,415 (32.6)	233 (40.5)	12.39	.001
	Excessive (≥ 2,300)	6,216 (67.0)	5,898 (67.4)	318 (59.5)		

All complex sample analyses were performed including missing values treated as valid cases. CAD = Coronary artery disease; Non-CAD = No coronary artery disease; SBP = Systolic blood pressure; DBP = Diastolic blood pressure; FBS = Fasting blood sugar; HbA1c = Hemoglobin A1c; TC = Total cholesterol; TG = Triglyceride; HDL = High-density lipoprotein cholesterol; LDL = Low-density lipoprotein cholesterol; BMI = Body mass index. ^†^w= Weighted sample; ^‡^Rao–Scott χ^2^ test for complex sample design; ^§^Variables include missing data in the analysis.

**Table 3.** Factors Associated with Coronary Artery Disease in Adults with Metabolic Syndrome

Variables	B	SE	OR	95% CI	*p*
Sociodemographic characteristics					
Age (years) (reference: 19~49)					
50~59	1.61	0.76	5.02	1.12, 22.46	.035
60~69	1.87	0.87	6.48	1.16, 36.04	.033
≥ 70	2.05	0.87	7.77	1.40, 43.13	.019
Educational status (reference: ≤ Elementary school)					
Middle school	−0.84	0.54	0.43	0.15, 1.24	.117
High school	−0.35	0.49	0.71	0.27, 1.84	.476
≥ College	0.56	0.48	1.75	0.68, 4.50	.244
Income level (reference: Low)					
Middle	0.52	0.39	1.68	0.78, 3.64	.185
High	−0.28	0.67	0.76	0.20, 2.85	.680
Health-related characteristics					
SBP (mmHg) (reference: < 120)					
120~139	0.94	0.43	2.56	1.10, 5.95	.029
≥ 140	1.31	0.59	3.69	1.16, 11.78	.027
DBP (mmHg) (reference: < 80)					
80~89	−0.05	0.43	0.95	0.41, 2.20	.906
≥ 90	−0.81	0.77	0.45	0.10, 2.02	.295
FBS (mg/dL) (reference: < 100)					
100~125	0.29	0.55	1.34	0.45, 3.95	.595
≥ 126	−0.26	0.58	0.77	0.25, 2.43	.660
HbA1c (%) (reference: < 5.7)					
5.7~6.4	0.03	0.63	1.03	0.30, 3.55	.966
≥ 6.5	1.35	0.72	3.86	0.93, 15.98	.062
TC (mg/dL) (reference: < 200)					
200~239	1.30	0.60	3.65	1.13, 11.78	.030
≥ 240	1.71	0.87	5.54	1.00, 30.68	.050
TG (mg/dL) (reference: < 150)					
≥ 150	0.18	0.74	1.20	0.28, 5.09	.807
LDL (mg/dL) (reference: < 130)					
130~159	0.45	0.67	1.57	0.42, 5.81	.502
≥ 160	0.32	0.93	1.37	0.22, 8.56	.736
BMI (kg/m^2^) (reference: < 23.0)					
23.0~24.9	0.40	0.52	1.49	0.54, 4.10	.438
≥ 25.0	0.07	0.47	1.07	0.42, 2.71	.885
Current smoker (reference: No)	0.29	0.44	1.33	0.56, 3.16	.516
Physical activity level (reference: Low)					
Moderate	−0.54	0.37	0.59	0.29, 1.21	.147
High	1.43	1.16	4.20	0.43, 41.22	.218
Sitting time (hours) (reference: < 8)	−0.11	0.35	0.90	0.46, 1.77	.757
Depressive symptom (reference: No)	0.47	0.46	1.60	0.65, 3.95	.308
Total energy (kcal) (reference: < 2,000)	0.28	0.56	1.32	0.44, 3.95	.614
Protein intake (g) (reference: < 54.6)	−0.23	0.51	0.79	0.29, 2.14	.648
Fat intake (g) (reference: < 67)	1.04	0.81	2.82	0.57, 13.91	.204
Sodium intake (mg) (reference: < 2,300)	0.19	0.39	1.21	0.56, 2.61	.631
F = 25.18 (*p* < .001), R^2^ = .28					

All complex sample analyses were performed including missing values treated as valid cases. SE = Standard error; OR = Odds ratio; CI = Confidence interval; SBP = Systolic blood pressure; DBP = Diastolic blood pressure; FBS = Fasting blood sugar; HbA1c = Hemoglobin A1c; TC = Total cholesterol; TG = Triglyceride; LDL = Low-density lipoprotein cholesterol; BMI = Body mass index.

## Data Availability

The data that support the findings of this study are available from the corresponding author upon reasonable request.
